# A missense mutation in a patient with developmental delay affects the activity and structure of the hexosamine biosynthetic pathway enzyme AGX1

**DOI:** 10.1002/1873-3468.13968

**Published:** 2020-11-18

**Authors:** Xiping Chen, Olawale G. Raimi, Andrew T. Ferenbach, Daan M.F. van Aalten

**Affiliations:** ^1^ Division of Gene Regulation and Expression School of Life Sciences University of Dundee Dundee UK

**Keywords:** enzyme mutation, neurodevelopment, *O*‐GlcNAcylation, pathogenesis, protein structure

## Abstract

*O*‐GlcNAcylation is a post‐translational modification catalysed by *O*‐GlcNAc transferase (OGT). Missense mutations in *OGT* have been associated with developmental disorders, OGT‐linked congenital disorder of glycosylation (OGT‐CDG), which are characterized by intellectual disability. OGT relies on the hexosamine biosynthetic pathway (HBP) for provision of its UDP‐GlcNAc donor. We considered whether mutations in UDP‐*N*‐acetylhexosamine pyrophosphorylase (UAP1), which catalyses the final step in the HBP, would phenocopy OGT‐CDG mutations. A *de novo* mutation in *UAP1* (NM_001324114:c.G685A:p.A229T) was reported in a patient with intellectual disability. We show that this mutation is pathogenic and decreases the stability and activity of the UAP1 isoform AGX1 *in vitro*. X‐ray crystallography reveals a structural shift proximal to the mutation, leading to a conformational change of the *N*‐terminal domain. These data suggest that the UAP1^A229T^ missense mutation could be a contributory factor to the patient phenotype.

## Graphical abstract

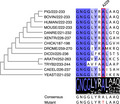

## Abbreviations

**DDD**, Deciphering Developmental Disorders Study

**GFAT**, glucosamine‐fructose‐6‐phosphate aminotransferase 1

**GNA1**, glucosamine‐6‐phosphate *N*‐acetyltransferase

**HBP**, hexosamine biosynthetic pathway

**LoF**, loss‐of‐function

***O*‐GlcNAc** *, O*‐linked β‐*N*‐acetylglucosamine

**OGT** *, O*‐GlcNAc transferase

**OGT‐CDG**, OGT‐linked congenital disorder of glycosylation

**PD**, Parkinson’s disease

**PGM3**, phosphoglucomutase 3

**UAP1**, UDP‐*N*‐acetylhexosamine pyrophosphorylase

*O*‐linked β‐*N*‐acetylglucosamine (*O*‐GlcNAc) is a post‐translational modification that occurs on thousands of nuclear and cytoplasmic proteins. The dynamic cycling of *O*‐GlcNAc is regulated by a single pair of enzymes, the *O*‐GlcNAc transferase (OGT), which transfers the GlcNAc moiety from UDP‐GlcNAc to Ser/Thr of protein targets [1], and *O*‐GlcNAcase, which removes *O*‐GlcNAc from the modified proteins [2]. Protein *O*‐GlcNAcylation is involved in various physiological processes, such as transcription, nutrient sensing and cell signalling [3, 4, 5, 6, 7]. In *Caenorhabditis elegans*, *O*‐GlcNAc orchestrates metabolism through the insulin‐signalling pathway to maximize neuron regeneration in response to neuronal injury [8]. The *Drosophila* OGT homologue is encoded by *super sex combs*, which is required for the repression of Polycomb genes [9], a cluster of genes essential for the development of animals and plants by dynamically regulating chromatin modification [10, 11, 12]. Deletion of either the *Ogt* or the *Oga* gene leads to embryonic lethality in mice [13, 14]. *O*‐GlcNAcylation is involved in neurodegenerative diseases, such as Alzheimer's disease [15], amyotrophic lateral sclerosis [16] and Parkinson’s disease (PD) [17, 18]. Recently, dysregulation of *O*‐GlcNAcylation caused by *OGT* gene mutations has been associated with a developmental disorder termed OGT‐linked congenital disorder of glycosylation (OGT‐CDG) with patients characterized by intellectual disability and developmental delay [19, 20, 21, 22, 23].

**Fig. 1:**
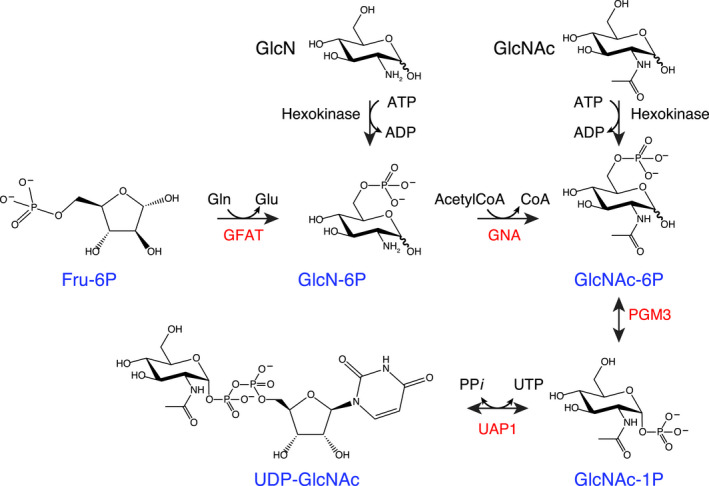
The eukaryotic HBP. The four main enzymes of the pathway are coloured in red, and metabolites generated by the HBP are coloured in blue. Reversible and irreversible reactions are indicated with double‐ and single‐headed arrows, respectively

UDP‐GlcNAc, the substrate of OGT, is the end product of the hexosamine biosynthetic pathway (HBP) (Fig. 1) that is present in all three kingdoms of life [24, 25, 26]. The first and rate‐limiting step of the HBP is catalysed by glucosamine‐fructose‐6‐phosphate aminotransferase 1 (GFAT) which transfers an amino group from glutamine to fructose‐6‐phosphate to produce glucosamine‐6‐phosphate. Glucosamine‐6‐phosphate then turns into GlcNAc‐6‐phosphate by the action of glucosamine‐6‐phosphate *N*‐acetyltransferase (GNA1). The third step is catalysed by phosphoglucomutase 3 (PGM3) that converts GlcNAc‐6‐phosphate into GlcNAc‐1‐phosphate. Finally, the production of UDP‐GlcNAc is reversibly catalysed by UDP‐*N*‐acetylhexosamine pyrophosphorylase (UAP1) which uses GlcNAc‐1‐phosphate and UTP as substrates in the forward reaction to produce UDP‐GlcNAc and pyrophosphate.

There are several lines of evidence that HBP is essential for development. *GFAT* gene knockout cells cannot survive without supplementation of the enzyme product, GlcNAc [27]. *GFAT* gene mutations have been linked to congenital myasthenic syndromes [28], a group of conditions owing to the defect of signal transmission from nerve cells to muscles [29]. *GFAT* patients displayed reduced levels of GFAT protein and *O*‐GlcNAcylation [28]. Inactivation of GNA1 in mouse embryonic fibroblasts led to defects in UDP‐GlcNAc biosynthesis, *O*‐GlcNAc modification and cell proliferation [30]. In addition, mutations of the *PGM3* gene that resulted in compromised enzymatic activity were identified as a cause of immunodeficiency, skeletal dysplasia and neurocognitive impairment [31, 32, 33]. A study in *Drosophila* showed that mutation of *nesthocker*, the orthologue of the *PGM3* gene, resulted in decreased levels of cytoplasmic UDP‐GlcNAc, disrupted protein *O*‐GlcNAcylation and finally blocked mesodermal development [34]. In *Drosophila,* the *UAP1* gene orthologue *mummy* modulates decapentaplegic (Dpp) signalling in the embryonic epidermis and is required for epithelial morphogenesis and nervous system development [35, 36, 37]. These data collectively suggest a link between the perturbation of the HBP, protein *O*‐GlcNAcylation and neurodevelopment.

The human *UAP1* gene encodes two different isoforms, named AGX1 and AGX2, with AGX1 being more abundant in testis and AGX2 in somatic tissues [38]. The two isoforms differ by a 17‐amino acid insertion in the *C* terminus of AGX2. Compared to AGX2, AGX1 has similar efficiency in producing UDP‐GlcNAc and 50 times more efficiency in producing UDP‐GalNAc [39]. Structural characterization revealed that both AGX1 and AGX2 comprise three domains, that is *N*‐terminal, catalytic and *C*‐terminal domain, and the 17‐amino acid insertion in the *C*‐terminal domain of AGX2 ablated oligomeric assembly [40]. *UAP1* genes, designated *glmU* in bacteria, have been reported to be essential in both prokaryotes and eukaryotes [41, 42]. For instance, inactivation of GlmU thermosensitive mutant in *Escherichia coli* resulted in a 37% reduction in peptidoglycan content and cell lysis [43]. The fungal *UAP1* gene orthologue *UAP* is essential in both *Saccharomyces cerevisiae* and *Aspergillus fumigatus* [44, 45]. Because the action of OGT depends on the steady supply of cytoplasmic UDP‐GlcNAc through the HBP, we considered whether *UAP1* gene mutations would phenocopy OGT‐CDG mutations.

As a clinical genetics programme, the Deciphering Developmental Disorders Study (DDD) was recently established to investigate undiagnosed developmental disorders using exome sequencing and array‐based detection of chromosomal rearrangements [46]. A *de novo* heterozygous mutation in the *UAP1* gene (NM_001324114:c.G685A:p.A229T, DDD entry: https://decipher.sanger.ac.uk/ddd/research‐variant/c9774b30226f59f8c1f79a7578fe5fc3/overview), which encodes a missense variant UAP1^A229T^, has been identified in a patient from the DDD study with reported cranial and skeletal abnormalities and intellectual disability. Here, we investigate possible effects of the A229T mutation on AGX1 using various *in vitro* approaches. First, clinical bioinformatics tools predict that the mutation is likely to be pathogenic, while biochemical analysis with recombinant AGX1^A229T^ reveals that the A229T mutation causes a reduction of protein thermal stability. Compared to wild‐type AGX1, AGX1^A229T^ has lower activity in producing UDP‐GlcNAc. X‐ray crystallographic structural characterization demonstrates that the A229T mutation lies proximal to the active site. The mutation induces local structural shift which weakens the hydrogen bond network connecting the *N*‐terminal and catalytic domain, leading to conformational changes of the *N*‐terminal domain that explains changes in catalytic activity. Together, these *in vitro* data suggest that the UAP1^A229T^ missense mutation could contribute to the patient phenotype.

## Materials and methods

### Construct cloning, protein expression and purification

The construct with full‐length AGX1 (UniProt Q16222) cloned into pGEX6P4 (expressing GST‐tagged protein) was obtained from our lab stock [47]. The A229T mutation was introduced using site‐directed mutagenesis with forward primer ggtggtctttatcggAcacttgcagcccag and reverse primer ctgggctgcaagtgTccgataaagaccacc. All constructs were verified by sequencing.

*E. coli* (DE3) pLysS was used for recombinant expression of *N*‐terminally GST‐tagged AGX1^wt^ and AGX1^A229T^. Protein expression was induced at 18 °C by addition of 200 µm IPTG when absorbance at OD_600_ reached 0.6 and incubated for a further 16 h. Cells were harvested by centrifugation at 4200 r·min^−1^ at 4 °C for 30 min and then resuspended in lysis buffer (100 mm Tris, 150 mm NaCl, 0.5 mm TCEP pH 7.5, 0.1 mg·mL^−1^ lysozyme, 0.1 µg·mL^−1^ DNase, 1 mm benzamidine, 0.2 mm PMSF and 5 µm leupeptin). Cells were lysed using French Press with a pressure of 50 000 psi. Cell lysate was then spun down at 20 000 r·min^−1^ at 4 °C for 30 min in an Avanti J‐25 centrifuge (Beckman). The supernatant was incubated with Glutathione‐Sepharose beads (GE Healthcare) at 4 °C for 2 h, and beads were then washed with 4–5 column volumes of lysis buffer. Bound protein was cleaved overnight from GST beads by PreScission protease at a final concentration of 0.5 µm. Protein was pooled then concentrated to 5 mL before loading onto a pre‐equilibrated Superdex 200 26/60 preparative column (AKTA Prime system used; GE Healthcare). Fractions containing pure protein were pooled and concentrated to 0.5 mL using a 50 kDa 20 mL Vivaspin concentrators. Protein was quantified using NanoDrop and then flash‐frozen in liquid nitrogen.

### Differential scanning fluorimetry assay

Assays were performed with 2 µm protein (1 mg·mL^−1^), 5 × SYPRO Orange dye (Sigma) and 1 mm ligand in the assay buffer (50 mm Tris pH 7.5, 150 mm NaCl, 2 mm MgCl_2_ and 0.5 mm TCEP) in a total volume of 25 µL. Fluorescence (λ_ex_ = 530 nm, λ_em_ = 560 nm) was monitored with a Bio‐Rad (CFX Connect™) real‐time system, while system temperature was increased from 20 to 90 °C in 1 °C increments. Data were fitted to Boltzmann sigmoidal curve using graphpad prism® 6 to obtain *T*_m_, which is the inflection point of the curve. Assays were performed with six biological replicates, and each biological replicate contained three technical replicates.

### Enzymatic activity assay

A coupled enzyme assay was deployed to study steady‐state kinetics. In the assay, AGX1^wt^ utilizes UTP and GlcNAc‐1P to produce UDP‐GlcNAc and pyrophosphate; pyrophosphate is then degraded by pyrophosphatase (coupling enzyme) into inorganic phosphate. BIOMOL Green® reagent (0.03% w/v malachite green, 0.2% w/v ammonium molybdate and 0.5% v/v Triton X‐100 in 0.7 N HCL) was used to detect the inorganic phosphate produced [48]. To determine Michaelis–Menten parameters, the assay was performed in triplicate with each reaction in a total volume of 100 µL buffer containing 50 mm Tris, pH 7.5, 10 mm MgCl_2_, 10% (v/v) glycerol, 1 mm DTT, 0.05 units pyrophosphatase and 20 nm AGX1, and one of the two substrates was kept at an excess concentration (500 µm), while the other one varied from 0 to 500 µm. Reactions were initiated by the addition of 20 nm AGX1^wt^/ AGX1^A229T^ and then incubated at room temperature for 10 min. Reactions were terminated by the addition of 100 µL BIOMOL Green® reagent. The assay plate was kept at room temperature for 30 min to allow colour development. The absorbance at 620 nm was measured using a Spectra max 340 PC. The assay was performed with five biological replicates, and each biological replicate contained three technical replicates. The turnover of substrates was < 10% under all conditions tested. Data were analysed using graphpad prism® to calculate *K*_m_, *V*_max_ and *P*‐values.

An XBZ (xanthene‐based Zn) assay was developed to probe the activity of AGX1^wt^ and AGX1^A229T^ catalysing the reverse reaction. In the XBZ assay, the fluorescent probe XBZ is activated once it binds to UTP [49]. The high sensitivity and selectivity of XBZ for NTP over NDP‐sugars and sugar‐phosphates has been demonstrated previously [49, 50]. Since pyrophosphate is one of the substrates of AGX1 in the reverse reaction and, in addition to UTP, it also induces XBZ fluorescence [49], we showed that 50 µm pyrophosphate was thoroughly degraded into inorganic phosphate after incubation with 0.05 U pyrophosphatase at room temperature for 30 min (Fig. S5A). A UTP standard curve was obtained using a UTP standard that was purchased from Sigma (catalog no. U6625) and serially diluted in the buffer containing 50 mm Tris pH 7.5, 0.5 mm MgCl_2_, 2% (v/v) glycerol, 20 µm pyrophosphate, 40 µm UDP‐GlcNAc and 0.05 U pyrophosphatase in a total volume of 100 µL (Fig. S5B). Plates were placed at room temperature for 30 min to allow the development of background signals. For measuring the reverse reaction catalysed by AGX1^wt^ and AGX1^A229T^, assays were performed in mixtures containing 50 mm Tris pH 7.5, 2% (v/v) glycerol, 0.5 mm MgCl_2_, 40 µm UDP‐GlcNAc and 20 µm pyrophosphate in a total volume of 100 µL. Reactions were initiated by addition of 0.5 nm enzyme and then incubated at room temperature for 1 min. Reactions were terminated by boiling for 5 min. After cooling down to room temperature, reactions were then supplied with 0.05 U pyrophosphatase (in a volume of 5 µL) and incubated further at room temperature for 30 min to allow thorough degradation of unconsumed pyrophosphate. XBZ was prepared at 15 µm in a buffer containing 25 mm HEPES pH 7.5, 10 mm NaCl, 75 µm pyrocatechol violet and 50% (v/v) methanol. Two hundred microlitre XBZ solution was added to each assay, and fluorescence was measured using a Spectra max 340 PC with *λ*_ex_ and *λ*_em_ set as 485 and 530 nm, respectively. The turnover of both substrates was below 10%. Data analysis was performed using graphpad prism®.

### Crystallization and structure solution

Before setting up crystal trays, AGX1^A229T^ (10 mg·mL^−1^) was buffer exchanged into crystallization buffer containing 25 mm Tris, pH 7.5, which is similar to the crystallization buffer of wild‐type AGX1 [40]. Crystallization was set up in sitting drop format with each drop containing 0.3 µL reservoir solution and 0.2 µL protein. Crystals appeared in 0.2 m MgCl_2_, 0.1 m Tris, pH 8.5 and 30% PEG 4000 within 3 days. Crystals were flash‐frozen in liquid nitrogen and sent to the I24 beamline of the UK National Synchrotron (Diamond Light Source) for data collection. Data sets were indexed and integrated with *iMOSFLM* [51]. The phase problem was solved by molecular replacement using *MOLREP* [52] and the PDB 1JV1 [40] as a phase donor. The structure was refined using *REFMAC*5 [53] and built manually using coot [54]. pymol [55] was used to generate figures.

### Bioinformatics analysis

UniProt IDs of protein sequences used to construct the phylogenetic tree are: F1S210 (*Sus scrofa*), F1MJP7 (*Bos taurus*), Q16222 (*Homo sapiens*), Q91YN5 (*Mus musculus*), F1QZD3 (*Danio rerio*), F6SUV5 (*Xenopus tropicalis*), F1NFV9 (*Gallus gallus*), Q9Y0Z0 (*Drosophila melanogaster*), Q54GN5 (*Dictyostelium discoideum*), Q940S3 (*Arabidopsis thaliana*), Q386Q8 (*Trypanosoma brucei brucei*), Q18493 (*C. elegans*) and P43123 (*S. cerevisiae*). A phylogenetic tree was calculated by clustalx [56] and visualized using mega7 [57] with topology displayed. Protein sequences were aligned using jalview [58].

## Results

### UAP1 A229T mutation is potentially pathogenic

In diploid organisms, haploinsufficiency is a phenomenon in which a single copy of a functional gene is not sufficient to produce the normal/wild‐type phenotype. Since the patient is only heterozygous for the UAP1 A229T missense mutation (DDD entry: https://decipher.sanger.ac.uk/ddd/research‐variant/c9774b30226f59f8c1f79a7578fe5fc3/overview), we first investigated whether the *UAP1* gene is associated with haploinsufficiency. Haploinsufficiency score (HI index) is the predicted probability of a gene to be haploinsufficient and over 80% of protein‐coding genes in the human genome have been scored ranging from 0 (haploinsufficient) to 100% (haplosufficient) [59]. Based on the analysis of protein‐coding genetic variation in 60 706 humans, the loss intolerance (pLI) is computed to predict the probability of a gene to be loss‐of‐function (LoF) intolerant [60]. High pLI scores (pLI ≥ 0.9) indicate LoF intolerance, whereas low pLI scores (pLI ≤ 0.1) indicate LoF tolerance. The predicted HI and pLI of the *UAP1* gene are 42.14% and 0.91, respectively, which suggests that the *UAP1* gene is potentially haploinsufficient and LoF intolerant.

**Fig. 2:**
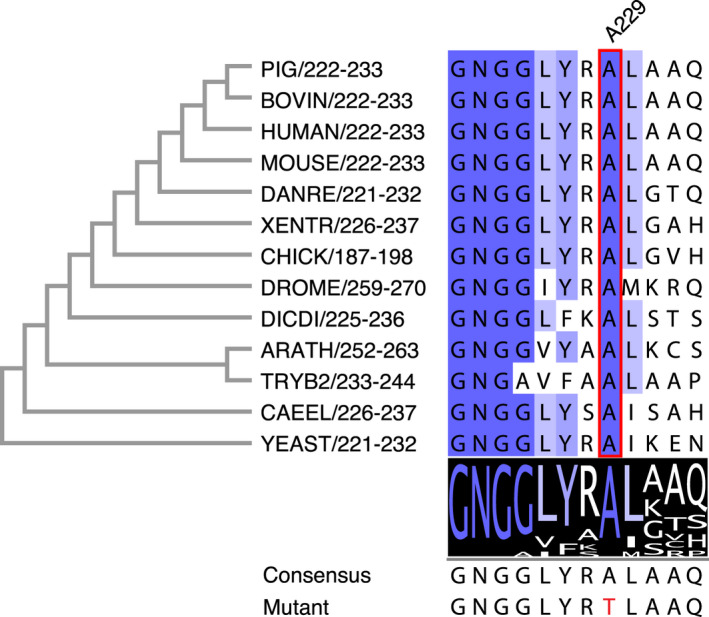
Sequence alignment of eukaryotic UAP1s. Sequences are named as organism abbreviation followed by sequence region. The phylogenetic tree was constructed based on full‐length proteins using clustalx [56] and then visualized with mega7 [57]. A229 is highlighted in a red box. DANRE, *Danio rerio*; XENTR, *Xenopus tropicalis*; DROME, *Drosophila melanogaster*; DICDI, *Dictyostelium discoideum*; ARATH, *Arabidopsis thaliana*; TRYB2, *Trypanosoma brucei brucei* (strain 927/4 GUTat10.1); CAEEL, *Caenorhabditis elegans*.

To investigate whether the UAP1 A229 mutation is tolerated in an evolutionary context, primary sequences of eukaryotic UAP1 orthologues were aligned. The data show that A229 is conserved in all eukaryotes (Figs 2 and S1), suggesting an important structural and/or functional role. In addition to sequence analysis, the UAP1 A229T mutation was predicted to be pathogenic by *in silico* tools, such as SIFT [61] (score = 0.03) and Polyphen‐2 [62] (score = 1) which evaluate effects of amino acid substitutions on proteins and MutationTaster [63] (*P* value = 1) which predicts the pathogenic potential of the DNA mutation. Together, these data suggest that the heterozygous UAP1 A229T mutation is potentially pathogenic.

### The A229T mutation decreases AGX1 stability

**Fig. 3:**
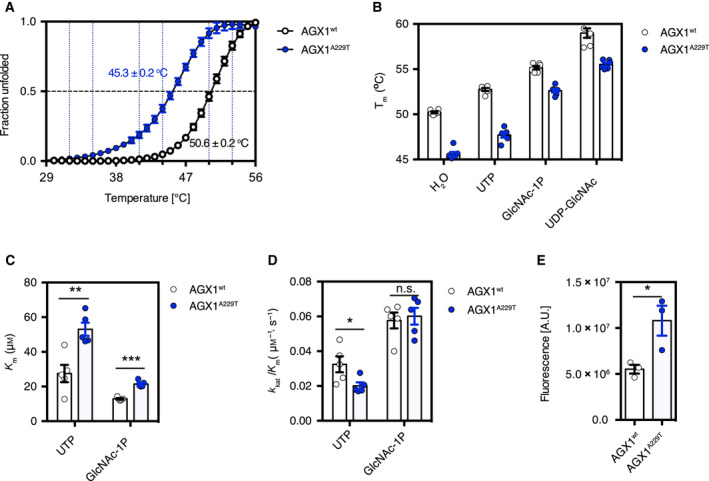
*In vitro* characterization of recombinant AGX1^A229T^. (A) Thermal denaturation curve showing unfolded fraction of recombinant AGX1^wt^ and AGX1^A229T^ as a function of temperature. Data were fitted to Boltzmann sigmoidal curve equation, with error bars representing SEM of six biological replicates. (B) Melting temperature (*T*_m_) of AGX1^wt^ and AGX1^A229T^ in the absence or presence of ligand. (C), (D) *K*_m_ and *k*_cat_/*K*_m_ of AGX1^wt^ and AGX1^A229T^ utilizing UTP and GlcNAc‐1P. Error bars represent SEM of five biological replicates. *P = 0.0382, **P = 0.0036, ***P < 0.001, *t*‐test. (E) Activity assay for the AGX1^wt^ and AGX1^A229T^ reverse reaction. Error bars represent SEM of three biological replicates. *P = 0.0359, *t*‐test.

We next investigated the possible effects of the A229T mutation on the biochemical properties of AGX1. AGX1^A229T^ was generated by site‐directed mutagenesis, and GST‐tagged AGX1^wt^ and AGX1^A229T^ were recombinantly expressed in *E. coli* and purified through glutathione‐affinity and size exclusion chromatography. During protein purification, it was noticed that AGX1^A229T^ was prone to forming aggregates in solution, which may explain the lower protein yield (1.0 mg·L^−1^ of culture) compared to AGX1^wt^ (4.5 mg·L^−1^ of culture). To examine whether this was the result of intrinsic AGX1^A229T^ stability, we analysed the thermal stability of AGX1^wt^ and AGX1^A229T^ using a differential scanning fluorimetry assay (DSF). This revealed that, compared to AGX1^wt^, AGX1^A229T^ was less stable as indicated by a reduction of the melting temperature (*T*_m_) by approximately 5.3 °C (Fig. 3A). DSF can also be used to observe protein–ligand interactions, which often increase the melting temperature [64, 65]. The melting temperatures of both AGX1^wt^ and AGX1^A229T^ were increased by incubation with the substrates/products, that is UTP, GlcNAc‐1P and UDP‐GlcNAc (Fig. 3B), suggesting that AGX1^A229T^ retains the ability to bind these substrates. Of note, AGX1^A229T^ was less stable than AGX1^wt^ regardless of which ligand was supplemented (Fig. 3B). Hence, these data suggest that the A229T mutation decreases AGX1 stability *in vitro*.

### The A229T mutation affects AGX1 activity

Given that A229 is conserved in eukaryotic UAP1 orthologues (Figs 2 and S1), and the A229T mutation resides in the catalytic domain, we next investigated the potential effects of the A229T mutation on the steady‐state kinetics of AGX1 catalysing the forward reaction. The reaction was monitored through a colorimetric assay using pyrophosphatase as the coupling enzyme, which hydrolyses the AGX1 pyrophosphate product leading to free inorganic phosphate that can be detected with Biomol Green [48]. The results show that, compared to AGX1^wt^, AGX1^A229T^ has a twofold increased *K*_m_ (*P* < 0.01, *t*‐test) towards both substrates (i.e. UTP and GlcNAc‐1P) and decreased catalytic efficiency in utilizing UTP (*P* = 0.0382, *t*‐test) (Fig. 3C,D). To enable comparison of AGX1^wt^ and AGX1^A229T^ activity in catalysing the reverse reaction, we used a xanthene‐based Zn (XBZ) fluorophore, which fluoresces upon binding of the reaction product UTP [49]. In this assay, AGX1^A229T^ is more active than AGX1^wt^ in consuming UDP‐GlcNAc and pyrophosphate (*P* = 0.0359, *t*‐test, Fig. 3E). Together, these data suggest that the A229T mutation affects AGX1 activity which may result in lower UDP‐GlcNAc levels *in vivo*.

### The A229T mutation induces structural changes

**Fig. 4:**
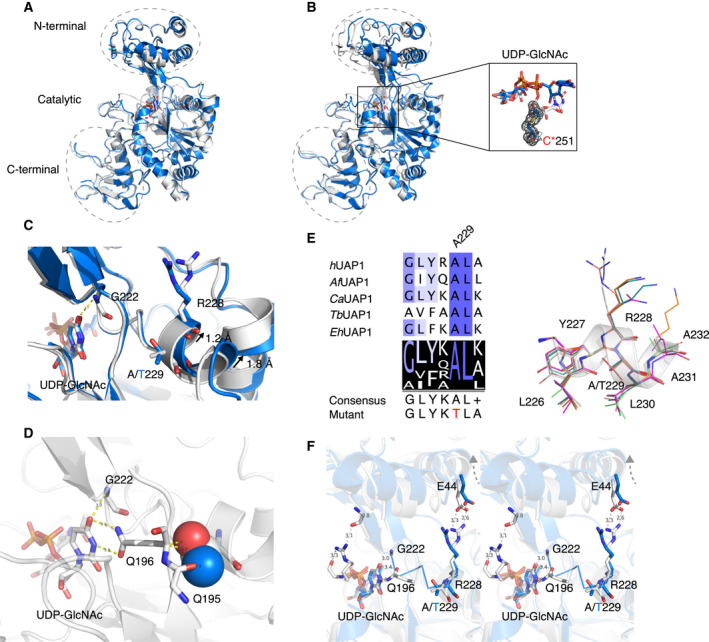
Structural analysis of AGX1^A229T^. (A) Superposition of AGX1^A229T^ A chain (blue cartoon) and AGX1^wt^ A chain (grey cartoon; PDB: 1JV1 [40]). (B) Superposition of AGX1^A229T^ B chain (blue cartoon) and AGX1^wt^ A chain (grey cartoon; PDB: 1JV1 [40]). UDP‐GlcNAc is highlighted as stick model in the active site of AGX1^wt^ A chain (C*_α_* blue) and AGX1^A229T^ B chain (C*_α_* grey). The electron density of carbamidomethylated Cys251 in AGX1^A229T^ B chain is shown as black mesh contoured to 2.5 σ. (C) Structure superposition showing the difference between AGX1^A229T^ A chain (blue cartoon) and the AGX1^wt^ B chain (grey cartoon; PDB: 1JV1 [40]) at the point of the A229T mutation. R228, A/T229, G222 and UDP‐GlcNAc are shown as a stick model. Structural shift induced by the mutation is indicated by the arrow. (D) Structure presentation of AGX1^wt^ showing the interaction between UDP‐GlcNAc, Q196 and A229. A229 is replaced by threonine and the side chain of the threonine is shown as sphere model. (E) Sequence and structure alignment of AGX1^A229T^ and eukaryotic UAP1 orthologues at the point of the A229T mutation. The helix in AGX1^A229T^ is shown in transparent cartoon representation with residues shown as a line model. (F) Stereoscopic view of the structural changes of AGX1 caused by the A229T mutation. The AGX1^A229T^ A chain (blue cartoon) is superposed to the AGX1^wt^ A chain (grey cartoon). The movement in the *N*‐terminal domain caused by the A229T mutation is indicated by the arrow. The main chain of M218 and G222 and the side chain of E44, R228 and A/T229 are shown as stick models. The segment which connects A229, UDP‐GlcNAc and the *N*‐terminal domain is shown as blue ribbon.

We next determined the crystal structure of AGX1^A229T^ to investigate the structural consequences of substituting the conserved A229 with a threonine. We were unsuccessful in obtaining the AGX1^A229T^ crystals using the published AGX1^wt^ crystallization condition [40]. However, crystals of AGX1^A229T^ in complex with UDP‐GlcNAc were obtained from screening commercial crystallization conditions, and synchrotron diffraction data were collected to 1.7 Å (Table 1). The structure was solved by molecular replacement using the published AGX1 structure (PDB: 1JV1 [40]) as a search model and refined to *R* = 0.187, *R*_free_ = 0.226. AGX1^A229T^ crystallized with two molecules in a *P*1 unit cell. Structural superposition of AGX1^A229T^ and AGX1^wt^ revealed conformational differences (pairwise RMSDs of the A and B chains 0.4–1.0 Å, Fig. S2) in the *N*‐terminal domain of both chains (Fig. 4A,B). In addition, the uridine moiety of UDP‐GlcNAc binding in the active site of the AGX1^A229T^ B chain points to the outside of the substrate binding pocket, which is different from the conformation of UDP‐GlcNAc binding in the active site of the AGX1^A229T^ A chain and AGX1^wt^ (Fig. 4B). We suggest that the conformational changes in the AGX1^A229T^ B chain are due to an alkylation of Cys251 in the active site that displaces UDP‐GlcNAc (Fig. 4B). We consider this alkylation to be artefactual as carbamidomethylation on multiple cysteines of both AGX1^wt^ and AGX1^A229T^ was observed with peptide mass fingerprinting (Table S1). Since our aim was to investigate the structural consequences of substituting the conserved A229 with a threonine, we focused on a detailed comparison of AGX1^wt^ A chain with its AGX1^A229T^ equivalent.

**Table 1:** Scaling and model‐building statistics of the AGX1^A229T^ crystal structure. Values in brackets are for the highest resolution shell.

Data collection	
Space group	P 1
Cell dimensions	
*a*, *b*, *c* (Å)	45.61, 64.57, 82.07
α, β, γ(^o^)	95.63, 101.09, 104.93
Resolution (Å)	29.26–1.70 (1.70–1.73)
*R*_merge_	0.024 (0.429)
*I*/*σI*	46.3 (2.6)
Completeness (%)	95.4 (90.9)
Redundancy	3.3 (3.2)
No. of reflections	93 239 (9102)
*R*_work_/*R*_free_	0.180/0.226
No. of nonhydrogen atoms	
Protein	7729
Ligand/ion	78
Water	715
*B* factors	
Protein	26.5
Ligand/ion	22.4
Water	32.9
RMSDs	
Bond lengths (Å)	0.011
Bond angles (°)	1.72
Ramachandran plot	
In preferred regions (%)	98.2
In allowed regions (%)	1.8
Outliers (%)	0.0
PDB code	6Z2F

In line with the decreased catalytic efficiency of AGX1^A229T^ in consuming UTP (Fig. 3D), the A229T mutation is in the middle of the α‐helix that coordinates the uridine moiety of UDP‐GlcNAc (Fig. 4C). In AGX1^A229T^ structure, there is a shift of 1.2 Å in the position of R228 (the residue next to A229) towards the *N* terminus and a shift of 1.8 Å in the *C* terminus of the helix encompassing the mutation to the opposite face of A229 (pushing effect) (Fig. 4C). In the A and B chains of AGX1^wt^, R228 interacts with E44 through two hydrogen bonds (3.0 and 3.1 Å in A chain; 3.3 and 2.6 Å in B chain), which contributes to the interaction between the *N*‐terminal and catalytic domain (Fig. S3). The R228‐E44 interaction is abolished in the AGX1^A229T^ structure caused by the position shift of R228 (Fig. 4F). The pushing effect is likely due to the bulkier side chain of threonine compared to that of alanine. In AGX1^wt^, the distances between the side chain of A229 and the main chain of Q195 and Q196 are 3.7 and 3.6 Å, respectively. However, In AGX1^A229T^ structure these distances are reduced to 2.7 and 2.2 Å, respectively, which is below the C‐O Van der Waals diameter (3.27 Å [66]). Since alanine has high helix propensity while threonine has high β‐sheet propensity [67], we then investigated whether the A229T mutation could cause distortion of this key active site α‐helix. We aligned the AGX1^A229T^ A chain with eukaryotic UAP1 orthologues at the point of the mutation. The results showed that, in the aligned sequences, even though there are sequence variations at positions apart from A229 and L230, the AGX1^A229T^ A chain retains the α‐helical conformation around position 229 (Fig. 4E). Together, the A229T mutation induced a local structural shift without disrupting the secondary structure of AGX1.

In a global structural view, the A229T mutation causes the *N*‐terminal domain of AGX1^A229T^ A chain to adopt an open/relaxed conformation compared to that of AGX1^wt^ (Fig. 4A,F). In AGX1^wt^ B chain, a distant interaction between Q112 in the catalytic loop and M218 in the *N*‐terminal domain is mediated by R169 through hydrogen bonds (3.3 and 3.1 Å). The Q112‐R169‐M218 interaction was observed in the AGX1^wt^ B chain but not A chain (Fig. S3), which probably indicates the interaction is dynamic in solution or catalysis. Along with the conformational change of the *N*‐terminal domain in the AGX1^A229T^ structure, is M218 shifted by 0.8 Å away from R169, weakening the Q112‐R169‐M218 interaction (Fig. 4F). Together, the data suggest considerable conformational changes induced by the A229T mutation.

## Discussion

UAP1 is the last enzyme in the HBP, reversibly converting UTP and GlcNAc‐1P to UDP‐GlcNAc and pyrophosphate. The steady supply of cytoplasmic UDP‐GlcNAc is not only required for normal cellular physiology [43, 44, 45], but also essential for development [28, 31, 32, 33]. *O*‐GlcNAcylation is one of the PTMs that depends on the steady supply of cytoplasmic UDP‐GlcNAc. There is accumulating evidence that dysregulation of *O*‐GlcNAcylation caused by mutations in the *OGT* gene is associated with a developmental disorder, termed OGT‐CDG [23]. This study was aimed at identifying other genes that are required for *O*‐GlcNAcylation that are mutated in patients with developmental disorders. In the recent DDD Study [46], a *de novo*, heterozygous *UAP1* gene missense mutation (NM_001324114:c.G685A:p.A229T) was identified in a patient with abnormalities of cranium, skeleton and nervous system. In this study, we demonstrated that UAP1 A229 is stringently conserved in eukaryotic UAP1 orthologues and the UAP1 A229T mutation is predicted to be pathogenic by clinical bioinformatic tools. Biochemical characterization revealed that, compared to AGX1^wt^, AGX1^A229T^ has decreased stability and activity in producing UDP‐GlcNAc while pyrophosphorolysis activity is increased. The stability and activity changes of AGX1 caused by the A229T mutation could collectively result in decreased UDP‐GlcNAc and *O*‐GlcNAcylation levels *in vivo*, giving a phenotype in the patient that resembles aspects of OGT‐CDG. However, experiments with CRISPR/Cas9 knock‐in cell lines or animal model systems are required to investigate this.

Structural analysis revealed that the A229T mutation induced both local and global structural changes. R228 shifted by 1.2 Å when A229 is mutated to threonine, followed by the disruption of the hydrogen bonds formed between R228 and E44. An allosteric inhibitor targeting *Trypanosoma brucei* UAP1 was discovered binding to the same site where R228 and E44 interact in human UAP1 (Fig. S4), and it was proposed that the inhibition was achieved by stabilizing the *N*‐terminal domain in a conformation that prevents UTP binding [47], suggesting that the R228‐E44 interaction in AGX1 could be contributing to regulation of activity. The R228‐E44 interaction contributes to the interaction between the *N*‐terminal and catalytic domains of AGX1^wt^, and disruption in the AGX1^A229T^ structure leads to a shift of the *N*‐terminal domain away from the catalytic domain. Accompanied by the shift of the *N*‐terminal domain, is the disruption of the hydrogen‐bonding interactions established through Q112‐R169‐M218 between the *N*‐terminal domain and the key catalytic loop. Given both the R228‐E44 and the Q112‐R169‐M218 interactions are human AGX1 specific [40, 68, 69] and AGX1 is over ten times more catalytically efficient than its fungal orthologues [45], they could be one of the factors that contribute to the high efficiency of AGX1, and disruption of them together in the AGX1^A229T^ structure could thus explain the stability and activity differences between AGX1^wt^ and AGX1^A229T^. Threonine is one of the common substitutions of alanine in nonsynonymous coding single nucleotide polymorphisms (ncSNPs) database [70], and alanine‐to‐threonine substitutions have been associated with human conditions, such as amyloid diseases [70] and PD [71]. It seems that the mutation intolerance of UAP1 A229 in the evolutionary context is not determined by the R228‐A229 interaction, as R228 is substituted to alanine in plants (Fig. 2), but more dependent on the interaction between the side chain of A229 and the main chain of Q195/Q196 (Fig. 4D). Together with the observation in fly models that the *Drosophila* UAP1 orthologue is required for epithelial morphogenesis and nervous system development [35, 36, 37], the UAP1^A229T^ missense mutation that results in considerable structure shifts that underpin the observed stability and activity changes of AGX1^A229T^ could be a contributory factor to the developmental delay observed in the patient.

Apart from being required for *O*‐GlcNAcylation, UDP‐GlcNAc is also the precursor for glycosylphosphatidylinositol anchor synthesis, heparan sulphate synthesis and *N*‐linked glycosylation. Dysregulation of these pathways has also been associated with developmental disorders [72]. For instance, *N*‐glycosylation is a common post‐translational modification in all three kingdoms of life that regulates protein stability, protein processing and function [73]. DPAGT1/GPT (Dolichol phosphate *N*‐acetylglucosamine‐phosphotransferase) catalyses the first committed step of *N*‐linked glycosylation, using dolichol phosphate and UDP‐GlcNAc as substrates [74]. Mutations in the *DPAGT1* gene, which result in alteration of either protein activity, stability or structure, have been identified as causing congenital disorders [75, 76, 77, 78]. Mutations of all the genes upstream of *UAP1* in the HBP have been identified as causing developmental disorders, and decreases in *O*‐GlcNAcylation levels are the common and predominant molecular phenotype observed in either patient‐derived cells, mouse or fly models [28, 31, 32, 33, 34]. *N*‐linked glycosylation levels were unchanged in cells carrying patient *GFAT* mutations [33] and in mouse embryonic fibroblasts carrying a *GNA1* gene disruption [30]; however, they were decreased in cells carrying patient *PGM3* mutations [33]. The pathways affected by the UAP1 A229T mutation require further dissection using appropriate models.

## Author contributions

DMFA conceived the study XC and ATF performed experiments. XC, OR and ATF analysed and interpreted the data. XC and DMFA wrote the manuscript with input from all authors.

## Funding

XC is funded by the China Scholarship Council studentship. DMFA is funded by a Wellcome Trust Investigator Award (110061).

## Supporting information


**Fig. S1.** Sequence alignment of eukaryotic UAP1s.
**Fig. S2.** Heatmap showing the RMSDs of superposition between the two chains of AGX1wt and AGX1A229T.
**Fig. S3.** Structural representation of AGX1^wt^ in complex with UDP‐GlcNAc.
**Fig. S4.** Structural superposition of AGX1^wt^ and *Tb*UAP1 crystal structures.
**Fig. S5.** Development of the XBZ assay to measure the reverse reaction catalysed by AGX1^A229T^.
**Table S1.** Analysis of AGX1^wt^ and AGX1^A229T^ carbamidomethylation by protein fingerprinting.

## Data Availability

The structure reported in this manuscript has been deposited in the Protein Data Bank with accession code 6Z2F. All remaining data are included within the manuscript and Supporting information.
