# Supplementary material for: A missense mutation in a patient with developmental delay affects the activity and structure of the hexosamine biosynthetic pathway enzyme AGX1
Source: FEBS Lett. 2020 Nov 18;595(1):110–22. doi: 10.1002/1873-3468.13968 (PMC7839538; doi:10.1002/1873-3468.13968)
Supplement: Supplementary file 1 — Fig. S1. Sequence alignment of eukaryotic UAP1s. Fig. S2. Heatmap showing the RMSDs of superposition between the two chains of AGX1wt and AGX1A229T. Fig. S3. Structural representation of AGX1wt in complex with UDP‐GlcNAc. Fig. S4. Structural superposition of AGX1wt and TbUAP1 crystal structures. Fig. S5. Development of the XBZ assay to measure the reverse reaction catalysed by AGX1A229T. Table S1. Analysis of AGX1wt and AGX1A229T carbamidomethylation by protein fingerprinting. [file FEB2-595-110-s001.zip › feb213968-sup-0006-Supinfo.docx]

**Supporting information**

**A missense mutation in a patient with developmental delay that affects AGX1 activity and structure**

Xiping Chen^1^, Olawale G. Raimi^1^, Andrew T. Ferenbach^1^ and

Daan M. F. van Aalten^1*^

^1^Division of Gene Regulation and Expression, School of Life Sciences, University of Dundee, Dundee, UK

**Contents**

**Supplementary Figures……………………………………………………………………….. S3**

**Supplementary Tables………………………………………………………………………… S8**

**Supplementary References……………………………………………………………………. S9**

**Supplementary Figures**

**Supplementary Figure S1. Sequence alignment of eukaryotic UAP1s.**

All sequences were retrieved from the UniProt database and aligned using the Jalview software(1). Sequences are named as organism abbreviation followed by sequence region**.** The phylogenetic tree was constructed using ClustalX(2) and then visualized with MEGA7(3). A229 is highlighted in a red box. Sequences are coloured based on percentage identity. DANRE, *Danio rerio*; XENTR, *Xenopus tropicalis*; DROME, *Drosophila melanogaster*; DICDI, *Dictyostelium discoideum*; ARATH, *Arabidopsis thaliana*; TRYB2, *Trypanosoma brucei brucei* (strain 927/4 GUTat10.1); CAEEL, *Caenorhabditis elegans*.

**Supplementary Figure S2. Heatmap showing the RMSDs of superposition between the two chains of AGX1^wt^ and AGX1^A229T^.**

WT_A, AGX1^wt^ A chain; WT_B, AGX1^wt^ B chain; A229T_A, AGX1^A229T^ A chain; A229T_B, AGX1^A229T^ B chain.

**Supplementary Figure S3. Structural representation of AGX1^wt^ in complex with UDP-GlcNAc.**

AGX1^wt^ (PDB 1JV1 (4)) is shown in cartoon model. UDP-GlcNAc and residues contributing to the interaction between the *N*-terminal and catalytic domain are shown as a stick model with gray carbons. Hydrogen bonds are indicated as yellow dashed lines.

**Supplementary Figure S4. Structural superposition of AGX1^wt^ and *Tb*UAP1 crystal structures.**

AGX1^wt^ (PDB 1JV1 (4)) is shown in gray cartoon representation with carbons of UDP-GlcNAc shown as gray sticks. *Tb*UAP1 (PDB 4BQH (5)) is represented as red cartoon and the carbons of the bound inhibitor are shown as green sticks. Structural changes in the *N*-terminal domain of *Tb*UAP1 induced by the inhibitor is indicated by the arrow.

**Supplementary Figure S5. Development of the XBZ assay to measure the reverse reaction catalysed by AGX1^A229T^.**

(A) XBZ fluoresces in the presence of UTP. The concentrations of UTP, pyrophosphate pyrophosphatase and inorganic phosphate were 5 µM, 50 µM, 0.05 U and 100 µM, respectively, where they were added. (B) UTP standard curve for the XBZ assay. UTP was serially diluted from 10 mM by a factor of two in the buffer containing 50 mM Tris pH 7.5, 0.5 mM MgCl_2_, 2% (v/v) glycerol, 20 µM pyrophosphate, 40 µM UDP-GlcNAc and 0.05 U pyrophosphatase in a total volume of 100 µl. Error bars represent the standard deviation of three determinations.

**Supplementary Table S1. Analysis of AGX1^wt^ and AGX1^A229T^ carbamidomethylation by protein fingerprinting**

| Cysteine | Peptide | Carbamidomethylation  (AGX1^wt^/AGX1^A229T^) | Peptide score (AGX1^wt^/AGX1^A229T^) |
| --- | --- | --- | --- |
| C158 | CIIPWYIMTSGR | Yes/No | 37/N.D. |
| C251 | GIWSIHVYCVDNILVK | Yes/Yes | 27/26 |
| C268 | FIGFCIQK | Yes/No | 21/N.D. |
| C275 | N.D. | N.D. | N.D. |
| C293 | TNPTEPVGVVCR | Yes/Yes | 39/45 |
| C431 | HALMSLHHCWVLNAGGHFIDENGSR | Yes/Yes | 58/65 |
| C464 | LKDANDVPIQCEISPLISYAGEGLESY  VADKEFHAPLIIDENGVHELVK | No/Yes | N.D./21 |

N.D. indicates not detected. Cysteine in the peptide sequences are highlighted in red.

Supplementary References

1. Waterhouse, A. M., Procter, J. B., Martin, D. M. A., Clamp, M., and Barton, G. J. (2009) Jalview Version 2--a multiple sequence alignment editor and analysis workbench. *Bioinformatics*. **25**, 1189–1191

2. Larkin, M. A., Blackshields, G., Brown, N. P., Chenna, R., McGettigan, P. A., McWilliam, H., Valentin, F., Wallace, I. M., Wilm, A., Lopez, R., Thompson, J. D., Gibson, T. J., and Higgins, D. G. (2007) Clustal W and Clustal X version 2.0. *Bioinformatics*. **23**, 2947–2948

3. Kumar, S., Stecher, G., and Tamura, K. (2016) MEGA7: Molecular Evolutionary Genetics Analysis Version 7.0 for Bigger Datasets. *Mol. Biol. Evol.* **33**, 1870–1874

4. Peneff, C., Ferrari, P., Charrier, V., Taburet, Y., Monnier, C., Zamboni, V., Winter, J., Harnois, M., Fassy, F., and Bourne, Y. (2001) Crystal structures of two human pyrophosphorylase isoforms in complexes with UDPGlc(Gal)NAc: role of the alternatively spliced insert in the enzyme oligomeric assembly and active site architecture. *EMBO J.* **20**, 6191–6202

5. Urbaniak, M. D., Collie, I. T., Fang, W., Aristotelous, T., Eskilsson, S., Raimi, O. G., Harrison, J., Navratilova, I. H., Frearson, J. A., van Aalten, D. M. F., and Ferguson, M. A. J. (2013) A Novel Allosteric Inhibitor of the Uridine Diphosphate N-Acetylglucosamine Pyrophosphorylase from Trypanosoma brucei. *ACS Chem. Biol.* **8**, 1981–1987
